# PEDIATRIC FRACTURES IN A TERTIARY PUBLIC HOSPITAL: WHAT ARE WE DEALING WITH?

**DOI:** 10.1590/1413-785220253301e285961

**Published:** 2025-02-03

**Authors:** Leonardo Lima de Almeida, Edgard Eduard Engel, Jose Batista Volpon

**Affiliations:** 1 Universidade de São Paulo, Ribeirão Preto Medical School, Department of Orthopedics and Anesthesiology - HCRP - FMRP-USP, Ribeirão Preto, SP, Brazil

**Keywords:** Child, Epidemiology, Fractures, Bone, Trauma, Physical, Child Health, Criança, Epidemiologia, Fraturas Ósseas, Trauma Físico, Saúde da Criança

## Abstract

**Objective:**

Orthopedic trauma is significant in modern society due to its incidence and its impact on healthcare and social interactions. Concerns include the risk of permanent sequelae affecting individual development and causing social stigma. Fractures, while not the most lethal lesion, may result in physical variable disability; publications show that about 30% of children experience fractures by skeletal maturity, primarily from low-energy trauma. This study aims to identify the fracture patterns in the immature skeleton at a tertiary-level public hospital.

**Methods:**

Individuals with skeletally immature fractures of the locomotor system, treated at a tertiary-level emergency unit from January 2016 to January 2020, were included. Data collected included social characteristics, trauma origin, fracture descriptors, and treatment modality. Age groups: infant, preschool, school-age, adolescent. Trauma energy is classified as low, moderate, or high.

**Results:**

A total of 926 cases were recorded in 505 patients, with a predominance of males. The most affected bones were the radius (29.5%), humerus (24.2%), and ulna (15.8%). The metaphysis was the most common location (46.7%), followed by the diaphysis (33.2%). Falls accounted for the largest portion, at 64.7%, with the majority (364) being low-energy trauma. High-energy trauma, such as pedestrian accidents and car accidents, represented 13.7%, and of these, 54.2% were polytraumatized.

**Conclusion:**

Fractures of the forearm persist as the most common, particularly at the distal third of the radius, with males being more exposed. Climatic seasonality and cultural traits such as soccer practice have little impact on the epidemiology of fractures. The results obtained in this investigation resemble those obtained by international literature. ***Level of Evidence III; Retrospective Cohort Study.***

## Resumo

**Objetivo:**

O trauma ortopédico é relevante na sociedade moderna devido à incidência e ao seu impacto na saúde e nas interações sociais. As preocupações incluem o risco de sequelas permanentes afetando o desenvolvimento individual e causando estigma social. Fraturas, embora não sejam as lesões mais letais, podem resultar em incapacidade física variável, alguns estudos mostram que cerca de 30% das crianças experimentam fraturas até a maturidade esquelética, principalmente devido a traumas de baixa energia. Esse estudo tem como objetivo identificar o padrão de fraturas do esqueleto imaturo de um hospital público de nível terciário.

**Métodos:**

Foram analisados indivíduos com fraturas do sistema locomotor, imaturos esqueleticamente, tratados em uma unidade de emergência de nível terciário entre janeiro de 2016 a janeiro de 2020. Os dados coletados incluíram características sociais, do evento traumático e da fratura; grupos etários infantil, pré-escolar, escolar, adolescente; energia do trauma classificada como baixa, moderada ou alta.

**Resultados:**

926 casos foram registrados em 505 pacientes, com predominância de homens. Os ossos mais afetados foram o rádio (29,5%), úmero (24,2%) e ulna (15,8%). A metáfise foi o local mais comum (46,7%), seguido pela diáfise (33,2%). Quedas representaram a maior parte (64,7%), sendo a maioria consideradas traumas de baixa energia. Trauma de alta energia, como acidentes com pedestres e acidentes de carro, representaram 13,7%, e destes, 54,2% foram politraumatizados.

**Conclusão:**

As fraturas do antebraço persistem como as mais comuns, particularmente no terço distal do rádio, com os homens estando mais expostos. Consideramos que a sazonalidade climática e os traços culturais, como a prática de futebol, têm pouco impacto na epidemiologia das fraturas. Os resultados obtidos nesta investigação se assemelham aos obtidos pela literatura internacional. **Nível de Evidência III; Estudo de Coorte Retrospectivo.**

## INTRODUCTION

Orthopedic trauma holds increasing significance in contemporary society, giving its rising incidence and significant impact on the healthcare system and social interactions. Emergency Department in U.S. register about 10 million visits on pediatric division per year, with 10 – 15% of musculoskeletal injuries.^1^ Although not typically fatal, certain fractures in children may cause permanent sequelae, which can affect individual development and lead to social stigma. In Great Britain around one-third of permanent sequelae in teenagers and young adults being related to orthopedic injuries, and in USA extremity injuries secondary to motor vehicle crash accidents in paediatric population counts for 30%.^2^,^3^

Approximately 30% of children and adolescents will sustain some fracture by skeletal maturity, with 60% resulting from low-energy trauma.^4^ In 2010 in the USA, approximately 1% of children experienced fractures requiring emergency care, incurring an average medical expense of US$7,000.00 per person, with higher costs for cases requiring surgical intervention.^5^ In addition to the economic impact, fractures disrupt family dynamics, as caregivers mobilize to aid in transportation, hygiene, and medical follow-up, with an average school absence of 14 days for upper extremity fractures and about 26 days for lower limb fractures.^6^ While short and long-term psychological implications have not been fully identified, motor limitations and increased dependency may exacerbate emotional stress within families, affecting the mental well-being of up to 25% of households.^7^

The epidemiology of fractures in the immature skeleton has consistent findings across international studies. Landin *et al.* (1983) noted a higher incidence among males, predominantly in fractures of the distal radius followed by hand fractures.^8^ Subsequent studies corroborated these observations, with approximately 80% of cases involving fractures of the upper limbs.^9^,^10^ However, there was some variation regarding the age of occurrence, with certain studies indicating a peak around 7 years old, while others reported around 11 years.^5^,^11^

Nevertheless, disparities may exist between Europeans and North American, and tropical countries due to climatic variations, cultural factors, and differing types of sports. Therefore, our study aims to investigate the specific characteristics of our population and fracture patterns to provide data for healthcare and contribute to formulation of public health policies.

## METHODS

We included skeletally immature individuals who presented fractures of the locomotor system treated at a public referral hospital from January 2016 to January 2020. The criterion used para characterize skeletal immaturity was the presence of the epiphyseal plate in the fractured bone. The inclusion criteria encompassed children or adolescents with fractures in one or more bones of the locomotor system (lower limbs, upper limbs, shoulder girdle, pelvic girdle) treated within 2 weeks after the fracture. Exclusion criteria comprised initial treatment performed at another institution, spine fractures, and incomplete data in the clinical records or radiographs. The study participants were exempted from signing the informed consent form following approval by the ethics committee (CAAE: 77303823.8.0000.5440).

History data were collected from the patient’s caregiver at the time of hospital admission and included characteristics as age, weight (kg), gender, traumatic event environment, seasonality, and trauma origin, including falls, direct trauma, sports activities, pedestrian, car accidents, and polytrauma. Fracture descriptors, such as the affected bone, side, bone topography (epiphysis, metaphysis, diaphysis), exposure of the fracture focus to the external environment, associated injuries, and treatment modality (surgical or non-surgical) were obtained from physical examination and radiographs.

Age groups were classified according to Landin et al. (1983) in the following categories: infant (0 to 1 year and 11 months old), preschool (2 years to 6 years and 11 months old), school-age (7 years to 11 years and 11 months old), adolescent (12 years old and above). Trauma energy was categorized as follows: low falls less than 50 cm), moderate (falls between 50 cm and 2 m), high (falls above 2 m).^8^

### Statistical Analysis

Descriptive and inferential statistics were conducted. The variables of interest were assessed using Student’s t-test for mean comparison, Pearson’s chi-square test, Pearson’s chi-square test with Bonferroni correction, and Fisher’s exact test for association. A significance level of 5% was adopted to all tests. Data analysis was performed using IBM SPSS statistical software (version 26.0, IBM Corporation, Armonk, New York, USA).

## RESULTS

**Figure 1:**
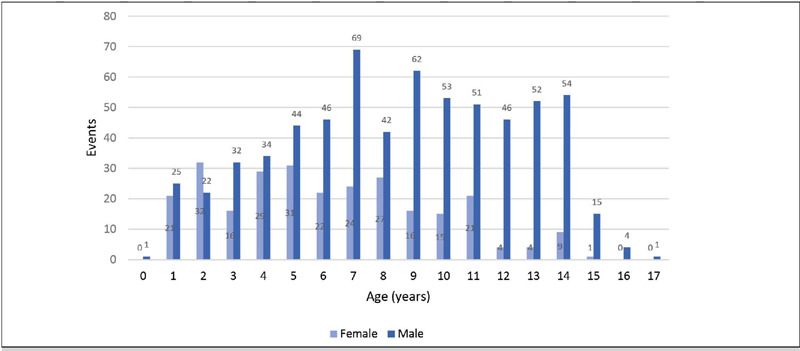
Fracture occurrence according to age demonstrates a predominance of males. After 5 years old this discrepancy becomes more increasingly evident. In boys, the significant occurrence of fractures extends up to 14 years of age and then declines. Conversely, for girls, this decline occurs around 11 years of age.

A total of 926 fractures were documented in 505 patients, with males comprising the majority (70.4% of occurrences). The overall mean age was 7.8 years (SD 3.8), with males having a mean age of 8.5 years and females 6.2 years (p < 0.001) (Figure 1).

**Figure 2:**
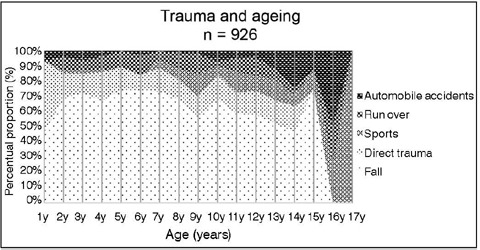
Types of trauma according to age. Overall, fractures resulting from falls were predominant. Fractures related to sports activities and car accidents were more common in older groups. Fractures caused by pedestrian accidents and direct trauma remained consistent until around 15 years of age.

Falls accounted for the largest cause, comprising 64.7% of the cases, with the majority (364) being falls of less than 50.0 cm, categorized as low-energy trauma (p<0,001). High-energy trauma, including pedestrian accidents and car accidents, accounted for 13.7% of cases with 54.2% of these resulting in polytrauma (Table 1). Regarding the primary mechanisms of trauma, falls emerged as the predominant cause across all ages examined, except at 16 and 17 years of age. Sports activities started to become apparent only from the age of 6 years. Incidences of car accidents remained relatively stable until around the age of 9, after which they began to rise proportionally. Accidents involving direct trauma remained constant at all ages studied, except for individuals aged 15 and older (Figure 2).

**Table 1:** Types of trauma and fractures.

		Total	%
Cause	Fall	592	63,9
	Run over	51	5,5
	Automobile accidents	78	8,5
	Daily activities	205	22,1
Total		926	100

**Figure 3:**
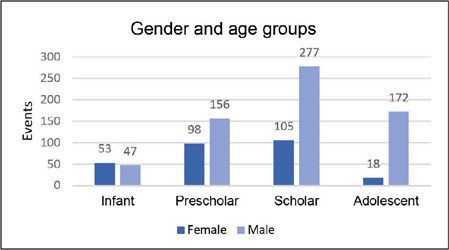
Distribution of fractures by age groups and gender.

**Figure 4:**
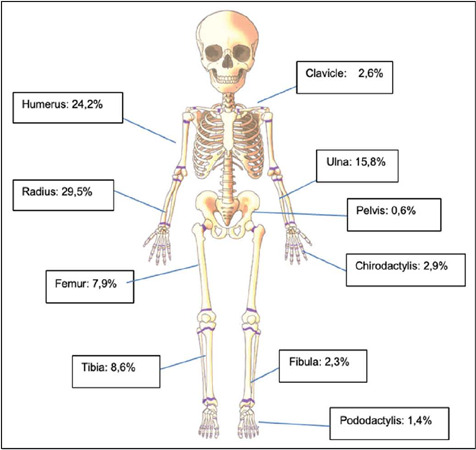
Percentage distribution of fractures. Source: Science Photo Library.

Fracture events were distinguished based on the gender of the patient and age group. It was observed that males in the preschool, school-age, and adolescent groups experienced a higher number of traumatic events compared to females (p < 0.001) (Figure 3). The most affected bones were the radius (29.5%), humerus (24.2%), and ulna (15.8%) (p < 0.001) (Figure 4). The metaphysis was the most common location (46.7%), followed by the diaphysis (33.2%). There was no predominance of fractures on the dominant side, with an equal distribution between right and left-handed individuals Additionally, no specific fracture pattern was identified with gender variation.

**Figure 5:**
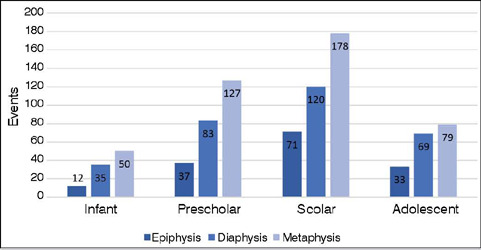
Distribution of fracture segments in relation to age group.

Regarding long bones, three primary classifications were made: epiphysis, metaphysis, and diaphysis. Metaphyseal fractures were the most common across all age groups, with highest prevalence in the preschool group (49%) and least prevalent in the adolescent group (41%). Diaphyseal fractures were more frequent in the adolescent group, accounting for 36% of cases, and less prevalent in the preschool group (14%). Epiphyseal fractures were most common in the school-age group, with 71 cases (18%), and less common in the infant population (12%) (Figure 5).

**Figure 6:**
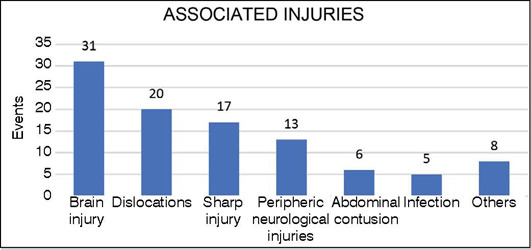
Associated injuries percentage distribution.

Associated injuries were observed in 7.1% of occurrences. The most common were traumatic brain injury (31%), dislocations (20%), lacerations (17%), and peripheric neurological injuries (13%) (Figure 6). Open fractures represented 7.7% of the sample, with 71 cases recorded. It was observed that 80% of fracture cases occurred during the school period. During this specific time, 53% of traumatic events occurred in the community environment, 37% at home and 9% in schools. During school vacations, 75% of events occurred in the community environment. Throughout the entire study period, 6.0% of fractures occurred in the school environment (Table 2).

**Table 2:** Distribution of events according to environment and seasonality.

Environment	Vacation	School Period	Total
Community	140	400	540
Home	45	281	326
School	0	60	60
Total	185	741	926

**Figure 7:**
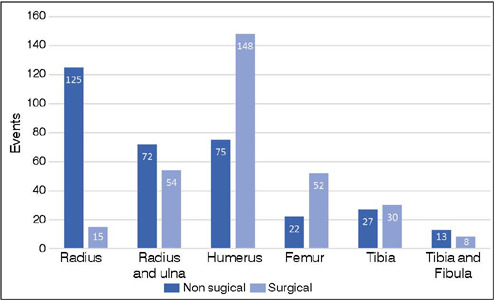
Surgical and non-surgical treatments for the long bones. The higher incidence of humerus fracture occurred at the supracondylar region.

Non-surgical treatment was indicated in 518 cases, representing approximately 55.9% of the sample. Among all conservatively treated fractures, only 51 (9.8%) resulted from high-energy trauma, indicating that almost 90% of conservatively treated cases originated from mild to moderate trauma. Femur fractures were predominantly managed surgically (70.2%), whereas isolated radius fractures were conservatively treated in 11% of cases (p < 0.001) (Figure 7). Surgical intervention was employed in 43% of combined radius and ulna fractures. Furthermore, there was a trend towards surgical management in humerus fractures (66.5%). A total of 228 cases were documented, and of these, approximately 155 were supracondylar fractures, of which 70.9% received surgical treatment.

## DISCUSSION

This study shows that forearm fractures in children persist as the most common injury, particularly highlighting the distal third of the radius, with males being more exposed than females. Simple falls proved to be the most common trauma mechanism, generally of low energy. We consider that climatic seasonality and cultural traits such as soccer practice have little impact on the epidemiology of fractures. The results obtained in this investigation resemble those obtained by authors in other countries^10^,^12^. However, our sample suggests a younger epidemiological peak, with the school-age group being more susceptible to traumatic events (41.3% of cases), which contrasts with studies where the highest incidence of fractures was in adolescence.^5^,^13^ Hedström et al. (2010) justify this profile due to incentives for physical activity and greater adherence to sports practices in the adolescent population.

We hypothesize some possible justifications for our data divergence from the international literature. The increased social interaction of children in this age group, as individuals who previously had their social circle limited to family members are now exposed to other peers and activities previously unexperienced.^14^ In this sense, it is worth noting the more friendly nature of latin american countries in interpersonal relationships. Another interesting aspect is progressive neuropsychomotor maturation, as around 6 years old, the child still has considerable difficulty in executing fine movements and from then on, begins to develop more complex and coordinated movements, based on an imitation pattern.^15^ Unfortunately, in Brazil, there is the perception that children from low-income families have a “shortened” childhood. Data from IBGE in 2022 showed that early school dropout, even at the elementary school age, was 8.5% by age 13; for children aged 13 and older, dropout rates reach 18%. The main reason, when asked, is the need to enter the workforce or disinterest in studies, as they do not see prospects.^16^

Simple falls during recreational activities were the most common mechanism of trauma, accounting for 64.7% of the analyzed cases. We believe that this will always be the most common mechanism of trauma for childhood fractures, as positioning the hand palm-down to avoid direct contact with the face, chest, and abdomen is an instinctive and reflexive mechanism for protection. In our sample, approximately 61% of falls (364 cases) were assessed as low-energy, representing just over one-third of the cases. The complexity of neuropsychomotor development, social interactions, and morphological changes in the pediatric skeleton make it difficult to develop effective protection policies for this mechanism of trauma. We believed that soccer, a prominent feature of Brazilian culture, could impact the number of lower limb fractures, specifically ankle fractures; however, this finding was not observed. Tibia and fibula fractures combined represented about 11% of occurrences, and the epiphyseal region, characteristic of ankle torsional events, accounted for 27.5% (28 cases). Even though soccer is a sport that requires skill in the lower limbs, both soccer and other ball sports can predispose to falls, and in this case, fractures of the upper limb prevail as the most common.

A higher incidence of supracondylar humerus fractures was observed, making it the second most affected bone, representing 24.2% of the casuistry. The literature presents conflicts in this aspect, with some studies corroborating this data,^5^ while others highlighting the clavicle, tibia, and fibula as more prevalent.^13^ We consider that our sample may present some bias, concentrating supracondylar humerus fracture cases due to excessive difficulties associated with this fracture and, consequently, more referrals for evaluation. For these cases, in about 44% of the occurrences, surgical treatment was proposed, with supracondylar humerus fractures and femoral shaft fractures being predominantly treated in this way (approximately 70% of cases). These numbers differ from some studies where conservative treatment is more prevalent, despite the progressive increase in surgical indications.^17^,^18^ There are questions about whether the trend toward surgical treatment results from the reception of more complex cases by the Institution or reflects a global trend of indicating surgeries more frequently for cases previously treated conservatively. Additionally, it is considered possible that families are more demanding regarding treatment outcomes, making the conservative approach less acceptable, which previously relied on bone remodeling and tolerance for slight residual deviations that did not compromise functionality.^19^

Sudden changes in the population’s lifestyle, such as the recent SARS-COVID-19 pandemic, have impacted the epidemiology of fractures, not only in the pediatric population. Social distancing, including the suspension of sports and leisure activities, resulted in a significant reduction (2.5 times) in the incidence of pediatric fractures, as shown by a recent study.^20^ However, this study period was not included in our survey.

The climatic seasonality analysis was based on the perception that warm weather encouraged young people to engage in recreational and sports activities. However, an analysis revealed a variable distribution of traumatic events throughout the year, with summer months not showing an increase in case incidence. As a matter of fact, only 22% of events occurred during the school vacation months, between December and February. Unlike countries in the northern hemisphere, where climatic seasons are more defined, in our country the climate is predominantly hot and dry, reaching uncomfortable levels of heat for much of the year. In places like the USA and Ireland, where summer months coincide with school vacations, there is a cultural expectation regarding outdoor activities, reflecting increases of up to 2.5 times in fracture incidence, a fact not observed in our sample.^21^

A similar study to the proposed here conducted in Colombia concludes that upper limb fractures continue to be the most incident injury (66% of cases), citing falls from own height as the most common trauma mechanism, and that males were the most affected.^22^ Colombian population have socio-cultural characteristics similar to Brazil, and no atypical epidemiological findings were observed when compared to European countries and the USA.

We conclude that seasonal and cultural aspects do not appear to have influence on the overall incidence of fractures, indicating that a child’s capacity for abstraction and creativity for leisure is universal, regardless of ethnicity, family financial support, and other social aspects.
